# Antibacterial biofilm efficacy of calcium hydroxide loaded on Gum Arabic nanocarrier: an in-vitro study

**DOI:** 10.1186/s12903-024-03941-3

**Published:** 2024-02-10

**Authors:** Alshafey Alsayed Mohamed, Dalia Mukhtar Fayyad, Mohamed El-Telbany, Dalia Abd-Allah Mohamed

**Affiliations:** 1 Department of Endodontics, Faculty of Dentistry, Suez Canal University, 4.5 Ring Road, Ismailia, 41522 Egypt; 2 Microbiology and Botany Department, Faculty of Science, Zagazig University, Zagazig, 44519 Egypt; 3 Department of Bioscience and Biotechnology, Graduate School of Bioresource and Bioenvironmental Sciences, Kyushu University, 744 Motooka, Nishi-Ku, Fukuoka, 819-0395 Japan; 4 Department of Endodontics, Faculty of Dentistry, Sinai University, Kantara, Ismailia, Egypt

**Keywords:** Calcium hydroxide, *Enterococcus faecalis*, Gum Arabic, Intra-canal medication, Nanocarriers, Quantitative PCR

## Abstract

**Background:**

An innovative intracanal medication formulation was introduced in the current study to improve the calcium hydroxide (Ca(OH)_2_) therapeutic capability against resistant *Enterococcus faecalis* (*E. faecalis*) biofilm. This in-vitro study aimed to prepare, characterize, and evaluate the antibacterial efficiency of Ca(OH)_2_ loaded on Gum Arabic (GA) nanocarrier (Ca(OH)_2_-GA NPs) and to compare this efficiency with conventional Ca(OH)_2_, Ca(OH)_2_ nanoparticles (NPs), GA, and GA NPs.

**Materials and methods:**

The prepared nanoparticle formulations for the tested medications were characterized using Transmission Electron Microscope (TEM) and Fourier-Transform Infrared Spectroscopy (FTIR). 141 human mandibular premolars were selected, and their root canals were prepared. Twenty-one roots were then sectioned into 42 tooth slices. All prepared root canals (*n* = 120) and teeth slices (*n* = 42) were divided into six groups according to the intracanal medication used. *E. faecalis* was inoculated in the samples for 21 days to form biofilms, and then the corresponding medications were applied for 7 days. After medication application, the residual *E. faecalis* bacteria were assessed using CFU, Q-PCR, and SEM. Additionally, the effect of Ca(OH)_2_-GA NP**s** on *E. faecalis* biofilm genes (*agg, ace, and efaA*) was investigated using RT-PCR. Data were statistically analyzed at a 0.05 level of significance.

**Results:**

The synthesis of NPs was confirmed using TEM. The results of the FTIR proved that the Ca(OH)_2_ was successfully encapsulated in the GA NPs. Ca(OH)_2_-GA NPs caused a significant reduction in the *E. faecalis* biofilm gene expression when compared to the control (*p* < 0.001). There were significant differences in the *E. faecalis* CFU mean count and CT mean values between the tested groups (*p* < 0.001) except between the Ca(OH)_2_ and GA CFU mean count. Ca(OH)_2_-GA NPs showed the least statistical *E. faecalis mean* count among other groups. SEM observation showed that *E. faecalis* biofilm was diminished in all treatment groups, especially in the Ca(OH)_2_-GA NPS group when compared to the control group.

**Conclusions:**

Ca(OH)_2_ and GA nanoparticles demonstrate superior anti*-E. faecalis* activity when compared to their conventional counterparts. Ca(OH)_2_-GA NPs showed the best antibacterial efficacy in treating *E. faecalis* biofilm. The tested NP formulations could be considered as promising intracanal medications.

**Supplementary Information:**

The online version contains supplementary material available at 10.1186/s12903-024-03941-3.

## Introduction

A tooth with nonvital pulp is considered an ideal habitat for microbial infection. Unfortunately, endodontic infections are polymicrobial, predominantly anaerobic, with the presence of some facultative bacteria [1]. *Enterococcus faecalis* (*E. faecalis)* is a gram-positive facultative anaerobic bacterium. It is often isolated from failed root canal treatment due to its resistance to conventional antibacterial medicaments and harsh intracanal conditions [2–4]. Furthermore, *E. faecalis* can form biofilms, enhancing its growth and persistence [5]. The ability of *E. faecalis* to cause pulpal and periapical diseases, such as apical periodontitis, is also attributed to virulence genes responsible for biofilm formation and attachment to root dentine surfaces, factors that may intensify collagen adherence. Additionally, *E. faecalis* can create biofilms between the dentine wall and root canal filling without being affected during the non-surgical root canal retreatment procedures [6, 7].

Accordingly, several antibacterial agents were used to control resistant and persistent infections like *E. faecalis*, and among them was calcium hydroxide Ca(OH)_2_ [8, 9] It is frequently used as an intracanal medicament because it can inhibit bacterial enzymes and control bacterial growth. However, Ca(OH)_2_ has some drawbacks, such as the need for prolonged treatment duration and cytotoxicity. Furthermore, *E. faecalis* has shown resistance to calcium hydroxide in different studies [9–12].

Generally, antibacterial drugs’ potential to treat infections brought on by multidrug-resistant (MDR) pathogenic bacteria has been decreased, causing a public health issue [13]. Thus, research is being done on innovative methods to eradicate persistent MDR bacteria. Regarding biomedical applications, research on nanoparticles has been encouraged, suggesting a viable alternative therapy to traditional antibiotics. Studies have demonstrated that the antimicrobial activity of nano-sized Ca(OH)_2_ is superior to that of conventional Ca(OH)_2_. This was explained by their capacity to penetrate *E. faecalis* biofilms considerably [14, 15]. In nano-size (less than 100 nm measurement), they can also infiltrate into the dentinal tubules more deeply than the traditional form [15, 16]. Other Studies were done to prepare and estimate the antibacterial biofilm efficiency of different calcium hydroxide formulations. Ca(OH)_2_ was successfully loaded on various polymers, and it was discovered that these formulations could reduce bacteria more effectively than traditional medications [17–20].

There has been significant interest in using natural polymers like Collagen, Gelatin, Fibrin, Chitosan, and Alginate to produce medications with polymeric NPs [19–21]. Besides, natural polymers have shown the advantages of intrinsic biocompatibility and biodegradability when used as nanocarriers [22]. Gum Arabic (GA), one of the natural polymers, is non-toxic, water-soluble, and vastly used in the stabilization of the food and pharmaceutical industries [23]. Its structure contains charged amine and carboxyl groups that can physically adsorb into the nanoparticle surface when used as a stabilizer [24, 25].

Gum Arabic was used as an oral hygiene agent to inhibit bacterial pathogens due to its antimicrobial, anti-inflammatory, and fungicide properties [26]. It was also shown that the nano-sized formulation of GA could improve its antimicrobial properties [27]. The current study suggested creating a new intracanal medication formulation by using GA nanoparticles for Ca(OH)_2_ encapsulation to combine the benefits of both Ca(OH)_2_ and GA antibacterial properties. It is unclear whether GA, nano GA, or Ca(OH)_2_ loaded on GA nanocarrier, when used as intracanal medicaments, have better anti-*E. faecalis* biofilm efficiency than Ca(OH)_2_ or nano Ca(OH)_2_. Therefore, the primary objective of this study was to evaluate in vitro the antibiofilm effect of Ca(OH)_2_ loaded on GA nano carrier against *E. faecalis* bacterial biofilm in infected root canals. The secondary objective was to compare the antibiofilm effect of Ca (OH)_2_, nano Ca(OH)_2_, GA, nano GA, and Ca(OH)_2_ loaded on nano GA against *E. faecalis* bacteria. The null hypothesis was the absence of a significant difference between the tested intracanal medications regarding their anti-bacterial effect against *E. faecalis*. The alternative hypothesis was that there would be a significant difference in the antibacterial efficacy of Ca(OH)_2_ with or without loading it in the GA nanocarrier.

## Materials and methods

The Preferred Reporting Items for Laboratory Studies in Endodontology (PRILE) 2021 standards [28] were followed when writing the paper for this laboratory study (Figure S1). This study was conducted with the approval of the Research Ethics Committee (REC) of the Faculty of Dentistry, Suez Canal University in Egypt (number 347/2021).

## Preparation of different formulations of intracanal medications

All intracanal medications used in this study were manufactured by Nano-Gate company (Nasr city,11,765, Cairo, Egypt).

### Preparation of calcium hydroxide nanoparticles (Ca(OH)_2_ NPs) powder

Ca(OH)_2_ nanoparticles were synthesized as per a previous study [29] using the ball mill technique. This involved milling conventional Ca(OH)_2_ powder (Loba Chemie, Mumbai, India) in a ball milling machine (planetary-ball-mill-pm-400, Retsch GmbH, Germany) for 10 h at a speed of 350 rpm with 3-minute intervals.

### Preparation of nano Gum Arabic (GA NPs)

Gum Arabic nanoparticles were synthesized based on a method from a previous study [27] with some modifications. Briefly, GA (Sigma Aldrich Chemical Co., Gillingham, UK) was gradually added to a chitosan solution (CH, Primex ehf, Siglufjordur, Iceland) in a 4:1 weight ratio (GA:CH). Subsequently, sodium tri-polyphosphate (TPP; Sigma Aldrich Chemical Co., Gillingham, UK) was added in a 1:3 weight ratio relative to chitosan. The mixture was then stirred continuously for 2 h to obtain GA NPs, with chitosan serving as the stabilizing agent [30, 31].

### Preparation of Ca (OH)_2_ loaded on GA NPs

Following the preparation of GA nanoparticles, Ca (OH)_2_ loaded on GA NPs was synthesized according to the previous studies [19, 27] by adding conventional calcium hydroxide (2.7 g) in the presence of GA NPs (2.7 g) with stirring for 30 min.

### Preparation of Ca(OH)_2_, Ca(OH)_2_ NPs, GA, GA NPs, and Ca (OH)_2_ loaded on GA NPs in gel form

To prepare gel forms for each of Ca(OH)_2_, Ca(OH)_2_ NPs, GA, GA NPs, and Ca (OH)_2_ loaded on GA NPs, 2.7 g of each material was mixed with 10mL of sterile distilled water (4–8 °C). The mixture was sonicated and stirred (SCILOGEX, RockyHill, USA) for 1 h then, 0.5 g of Hydroxypropyl Methyl Cellulose (Sigma Aldrich Chemical Co., Gillingham, UK) was gradually added under mild temperature with vigorous stirring. The resultant mixture was then cooled (2–4 ^0^C) to form a homogeneous gel.

## Characterization of prepared intracanal medications

### A Transmission Electron Microscope

(JEOL JEM-2100 high-resolution TEM, Tokyo, Japan), equipped with Selected Area Electron Diffraction (SAED) analysis was utilized to assess the size, shape, and distribution of the prepared nanoparticles (NPs). This analysis was conducted at an accelerating voltage of 200 kV, following the methodology described by Sharkawy et al. [27].

### Fourier-Transform Infrared Spectroscopy

(FTIR, Shimadzu Europa, Duisburg, Germany) was employed to evaluate the compatibility of prepared medications. This was achieved by observing the functional groups involved in stabilizing the synthesized nanoparticles, as per the guidelines in references [30–32].

## Evaluation of the effectiveness of the studied intracanal medications against *E. faecalis* biofilm (Fig. 1)

**Fig. 1:**
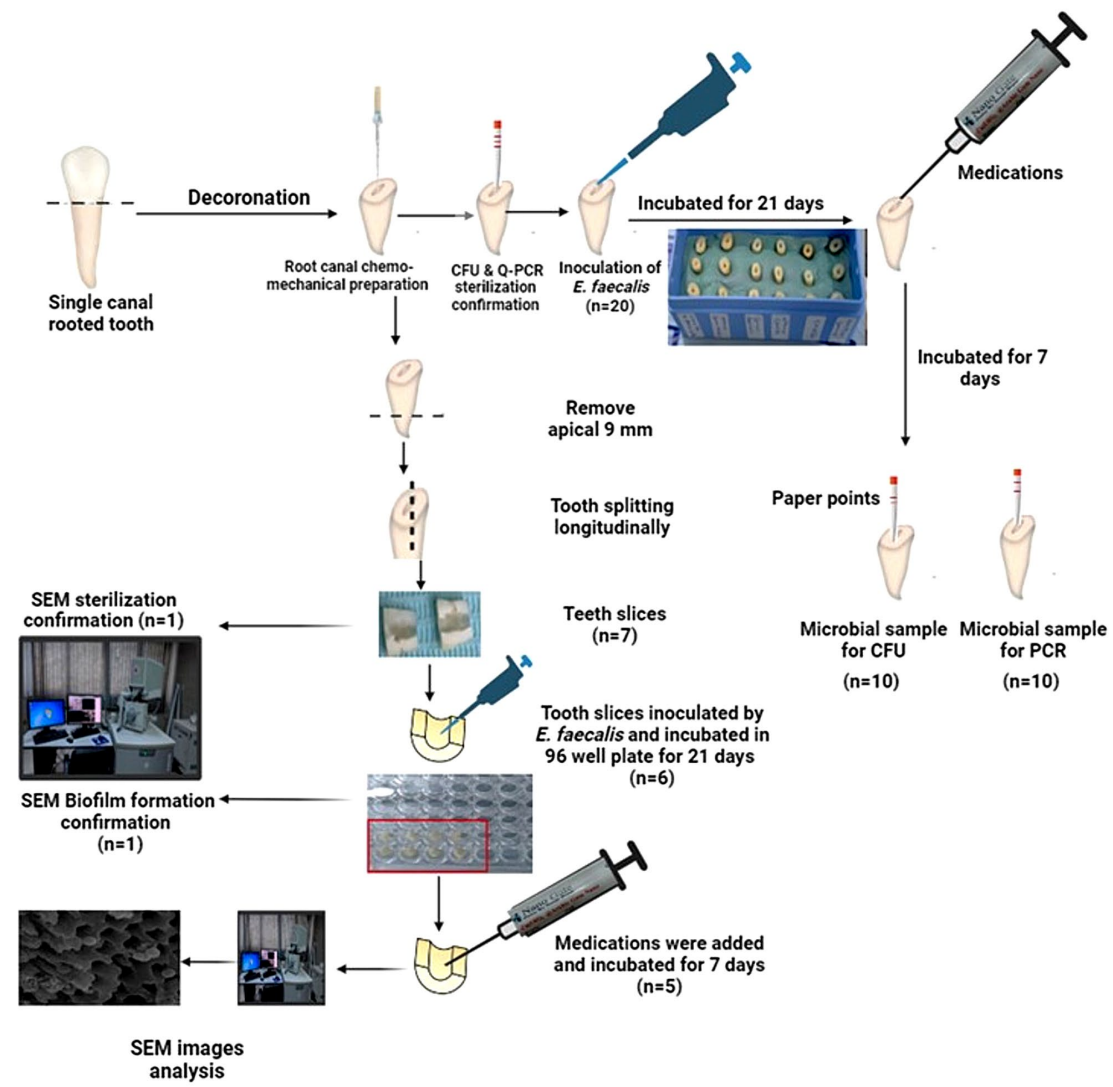
Schematic diagram showing the study design including the flow of teeth samples’ preparation (root canal model & teeth slices), sample size per each group (n), *E. faecalis* inoculation, medication placement, quantitative (CFU&Q-PCR) and qualitative (SEM) analysis

### Sample size calculation

The sample size used in the current study was calculated using G*Power software 3.1.9.6 (G Power; Franz Faul, University of Kiel, Germany) [33], and it was in accordance with a prior study [8].

(A) *Evaluation of antibiofilm efficiency of tested medicaments using Colony Forming Unit (CFU) and Quantitative Real-Time Polymerase Chain Reaction (Q-PCR) tests*:

To assess the antibiofilm effectiveness of Ca(OH)_2_ loaded on GA nanocarrier and compare it with Ca(OH)_2_, Ca(OH)_2_ NPs, GA, GA NPs, and Hydroxypropyl Methyl Cellulose (control group) using CFU and Q-PCR analysis, repeated measures ANOVA was employed. The study was designed to detect a 0.20 effect size with a power of 90% (1-β = 0.90), at a significance level of *p* < 0.05 and a partial eta squared of 0.04. This required a minimum total sample size of 114. Based on these calculations, the study included six groups (A0, A1, A2, A3, A4, A5), with each group comprising at least 20 samples. These were further divided into two subgroups (*n* = 10) for different evaluation methods, with 10 specimens for PCR and 10 for CFU analysis. Therefore, the total sample size selected for the study was 120 specimens.

(B) *Evaluation of antibiofilm efficiency of tested medicaments using Scanning Electron Microscopy (SEM)*:

For evaluation of Ca (OH)_2_ loaded on GA nanocarrier antibiofilm efficiency and to compare its antibiofilm efficiency with Ca (OH)_2_, nano Ca (OH)_2_, GA, nano GA, and Hydroxypropyl Methyl Cellulose (control group) using SEM. The minimum total sample size was 30 (30 tooth slices from 15 teeth) to detect an effect size of 0.4 and 80% power (1-β = 0.80) at a partial eta squared of 0.14 at a significance of 0.05 significance. Based on these calculations, the study had six groups (A0, A1, A2, A3, A4, A5), each represented by at least 5 samples. An additional 12 samples (from 6 roots) were included, two for each group. One sample per group was used to verify sample sterilization before bacterial inoculation, and another to confirm biofilm formation by SEM prior to medication application. Thus, each group ultimately comprised 7 samples.

### Selection of the teeth

A total of one hundred forty-one (*n* = 141) unidentified human mandibular premolars, freshly extracted for orthodontic reasons, were used in this study. Digital periapical radiographs were utilized for the preoperative assessment of these teeth, which were required to meet the following inclusion and exclusion criteria.

Inclusion criteria: Teeth had to be sound, single-rooted with a type I single canal (according to Vertucci’s classification [34]), and possess completely formed roots. Exclusion criteria: Teeth exhibiting external or internal root resorption, calcifications, hyper-cementosis, root canal variations, caries, or root fractures were excluded. The selected teeth were cleared of debris and calculus using an ultrasonic scaler (Miltex, Davies Drive, York) and then ultrasonically cleaned in distilled water for ten minutes. Teeth were subsequently immersed in 5.25% sodium hypochlorite (NaOCl, DEXA company for chemicals, Egypt) for 30 min to remove any soft tissue on the external root surface. This was followed by a final rinse with a 5% sodium thiosulfate solution (PIOCHEM, Egypt) to neutralize the sodium hypochlorite. Finally, the teeth were stored in normal sterile saline (0.9%) (Al Mottahedoon Pharma, Egypt) at room temperature until use.

### Teeth samples preparation

#### Root canal preparation

All the included teeth (*n* = 141) were prepared as follows: A rotary diamond disk (Sharp Inc, Luzern, Switzerland) mounted on a low-speed handpiece with water coolant was used to decoronate the teeth just below the cementoenamel junction, standardizing the root length to15 mm. The working length was established by subtracting 1 mm from K file (Micro Mega, Besancon, France) size #15, ensuring it was visible at the apical foramen. Canal patency and working length for each tooth were verified radiographically using K files of sizes #10 and #15. Root canals were prepared using elephant rotary (Katowice, Poland) Ni-Ti system (torque = 2.5 N.cm, speed = 350 rpm) employing a step-down technique till the master apical file (MAF) reached #30 taper 4%, following the manufacturer’s instruction. Irrigation involved 3mL of 3% NaOCl (DEXA company for chemicals, Cairo, Egypt) for three minutes after each file, administered via a 3mL plastic syringe with 30-gauge side-vented needles (Laigues, Vd, Switzerland). To remove the smear layer, the canals were rinsed with 3mL of a 17% ethylenediaminetetraacetic acid (EDTA, Prevest Denpro Limited Company, Jammu, India) solution for five minutes, followed by rinsing with 10 mL of 10% distilled water and 5% sodium thiosulfate (PIOCHEM, Cairo, Egypt) respectively, to neutralize any remaining EDTA or sodium hypochlorite effect. Of the samples, 120 randomly selected root samples had their external surfaces dual-coated with nail polish, and the apices were sealed with a light-cured resin composite (Tetric, Ivoclar Vivadent, Schaan, Liechtenstein).

#### Teeth preparation for Scanning Electron Microscopic (SEM) evaluation

To evaluate the effectiveness of various intracanal medications against bacterial biofilm using SEM, the remaining 21 prepared roots (*n* = 21) were used. These roots were horizontally sectioned to remove the apical portion (9 mm), resulting in standardized root segments of six mm in length. Subsequently, the root segments were longitudinally divided into two halves to create 42 semi-circular root samples. This splitting was performed using a saw with a 0.3 mm blade thickness (Isomet 4000, Buehler Ltd., Lake Bluff, USA) at 1000 rpm, with underwater cooling for precision and to prevent heat damage.

### Grouping of the teeth samples

All prepared root canals (*n* = 120) and teeth slices (*n* = 42) were divided into six groups according to the type of intracanal medication. Group A: Hydroxypropyl Methyl Cellulose (control), group B: Ca(OH)_2_, group C: nano Ca(OH)_2_, group D: Gum Arabic (GA), group E: nano GA and group F: Ca (OH)_2_ loaded on nano GA nanocarrier. Subsequently, each group was further divided into three subgroups according to different evaluation techniques. Ten root canal samples from each group were allocated for the colony forming unit test (CFU), another ten for the quantitative polymerase chain reaction test (Q-PCR), and seven teeth slices were used for SEM evaluation (Fig. 1).

#### Blind randomization of the prepared teeth samples

For blind randomization, each prepared root sample/slice was masked in an opaque envelope and coded by the allocator (resident dentist) using numerical identifiers (e.g., 1, 2, 3, 4, etc.). Similarly, the six syringes containing the tested intracanal medications were also masked and randomly assigned letter codes (A, B, C, D, E, F) by the allocator. Randomization of the samples was conducted using Microsoft Excel software. During the study, the operator selected the teeth sample and corresponding medicaments according to the predetermined randomization (e.g., 1-D,2-F) without knowledge of treatment type, Likewise, the evaluator was also blind to study groups, ensuring a double-blinded study design.

### Preparation of *Enterococcus faecalis* bacteria

The standard *E. faecalis* bacterial strain used in this study was sourced from the American Type Culture Collection (ATCC #29,212). Bile esculin azide agar (BEA; Sigma-Aldrich, St Louis, MO) served as the selective medium to *E. faecalis* purification.

#### Biofilm formation capacity test

The Congo red agar assay (CRA), as described in a previous study [35], was employed to confirm the biofilm-forming capability of the selected *E. faecalis* strain. CRA was prepared and autoclaved then cooled, and aseptically poured into sterile glass petri plates (Sigma Aldrich Chemical Co., Gillingham, UK). Three plates were designated for the assay, onto, each of which 50 µL of *E. faecalis* culture (O.D. = 0.5) was streaked. The plates were at 37 °C overnight. The following day, the appearance of black colonies with dry, crystalline consistency was indicative of biofilm formation, while non-biofilm producers were identified by the growth of pink colonies.

### Sterilization of the teeth samples and prepared medications

Each prepared root sample (*n* = 120) and slice (*n* = 42) was placed in a cryovial containing 500 µl of brain heart infusion (BHI; EL Nasr chemical co. Adwice, Egypt), and then autoclaved at 121 °C for 30 min to ensure sterilization. The sterilized teeth samples were vertically positioned in a sterile container specifically designed for this study, using a rubber base (Zeta Plus, condensation silicone, Zhermack, Rovigo, Italy) impression material for fixation. Additionally, the teeth slices were individually placed in a 96-well sterile microtiter plate (Sigma Aldrich Chemical Co., Gillingham, UK), one slice per well. These entire setups underwent a second autoclave cycle to guarantee sterility [36].

To confirm sterilization, different methods were employed. Samples incubated for 48 h at 37° C were randomly tested for sterility, using subcultures on blood agar. CFU and Q-PCR tests were conducted on randomly selected sterility samples (S0) using sterile absorbent paper points (size #30, taper 4%, Dentsply Maillefer, USA). Additionally, one tooth slice from each group (*n* = 6) was randomly chosen for SEM evaluation to further confirm sterilization. The prepared intracanal medicaments were sterilized before measuring antibacterial efficiency. The nanoparticles underwent γ-irradiation treatment, with a radiation dose of 25 kGy at a source temperature of 60 °C, in accordance with the European Pharmacopoeia [37].

### Bacterial (*E. faecalis*) inoculation in root canals and teeth slices

*E. faecalis* bacteria were cultured and cultivated overnight at 37 °C in a brain heart infusion broth (BHI; EL Nasr chemical co. Adwice, Egypt) on a rotary shaker at 150 rpm. The microbial growth was monitored by observing changes in turbidity after 24 h. A standard volume of 15µL of *E. faecalis* culture (O.D = 0.5) was inoculated into each root canal (*n* = 120), while 7.5µL of the same culture (O.D. = 0.5) was inoculated into each tooth slice (*n* = 36), using an insulin syringe (AdvaCare, Cheyenne, USA). To facilitate biofilm formation and bacterial invasion into the dentinal tubules, the samples were cultured for an additional 21 days [38]. The BHI medium was replaced every three days with sterilized culture medium (BHI) to ensure the continued viability of the bacteria [39].

### Conformation of biofilm formation at 21 days

Biofilm formation was quantitatively evaluated after 21 days for each group using Colony Forming Unit (CFU) and Q-PCR tests. Qualitative analysis was conducted using SEM evaluation. This was done to confirm that all samples had almost the same bacterial load before testing the intracanal medications. Any sample exhibiting extreme results was excluded and replaced with another to maintain consistency in the study.

#### Colony Forming Unit (CFU) assessment

Three sterile absorbent paper points (size #30; Dentsply Maillefer, USA) were employed to collect the microbiological samples (S1) from the root canals. Each paper point was inserted into the root canals for 60 s and gently pumped to collect the bacterial suspension from the main pulpal space. After collection, the paper points were placed into vials containing 0.25 mL of sterile saline solution. The vials were then vortexed for 15 s. Serial dilutions of samples were prepared, and 0.1 mL from each dilution was seeded onto bile Aesculin agar (Oxoid, Basingstoke, UK) plates. The plates were incubated at 37 °C for 24 h, after which the colonies were counted from the appropriate dilution and expressed as colony-forming units (CFU/mL).

#### Quantitative Polymerase Chain Reaction (Q-PCR) assessment

As previously described, microbiological samples (S1) were collected using three sterile absorbent paper points (size #30). These collected samples were then transferred into vials containing 0.25 mL of sterile saline solution. RNA isolation was performed in accordance with a prior study [40] utilizing the QIAamp RNeasy Mini kit (Qiagen, Hilden, Germany). Each sample, in a volume of 200 µl, was mixed with 600 µl of RLT buffer, which included 10 µl of β-mercaptoethanol per 1 mL. This mixture was then incubated at room temperature for 10 min. The lysate was combined with an equal volume of 70% ethanol, and the procedure followed the total RNA Purification protocol of the QIAamp RNeasy Mini kit.

For *E. faecalis* detection, species-specific oligonucleotide primers were used: 5’-GTT TAT GCC GCA TGG CAT AAGAG-3’ (forward primer) and 5’-CCG TCA GGG GAC GTT CAG-3’ (reverse primer). The Q-PCR reaction, with a total volume of 25 µl, included 10 µl of 2x HERA SYBR® Green RT-qPCR Master Mix (Willowfort, Birmingham, UK), 1 µl of RT Enzyme Mix (20 X), 0.5 µl of each primer at 20 pmol concentration, 5 µl of water, and 3 µl of RNA template. This reaction was conducted using a Q-PCR machine (Stratagene MX3005P, Agilent, Santa Clara, USA), and the Amplification curves and Cycle Threshold (CT) values were analyzed using AriaMx software (Agilent, Santa Clara, USA).

#### SEM qualitative evaluation

One tooth slice was randomly selected from each group (*n* = 6) for analysis. The selected slices were rinsed with sterile saline and then fixed in 2.5% glutaraldehyde for 2 h at 4 °C. Following fixation, the samples were washed again with sterile saline and dehydrated using a series of graded ethanol solutions. After dehydration, the samples were dried, gold-coated, and then imaged using a JEOL SEM (JSM 6510 lv, USA) at different magnifications (x1500, x3000) to inspect and validate the *E. faecalis* bacterial biofilm formation at various locations on each tooth slice.

### Application of the tested medications in root samples

After 21 days, the samples were flushed using 5 mL of sterile water to remove the inoculated broth. Subsequently, the prepared root canals (*n* = 20) and remaining teeth slices (*n* = 5) in each group were treated with their respective intracanal medications. This was done using insulin syringes (AdvaCare, Cheyenne, USA), administering 0.4 mL for each root canal and 0.1 mL for each tooth slice. The groups of the treatments were as follows: group (A): Hydroxypropyl Methyl Cellulose (control samples), group (B): 27% w/v Ca (OH)_2_, group (C): 27% w/v nano Ca (OH)_2_, group (D): 27% w/v Gum Arabic (GA), group (E): 27% w/v nano GA, and group (F): Ca (OH)_2_ (27%) loaded on GA nanocarrier (27% w/v). After treatment, the roots were coronally sealed with a sterile Teflon pellet and capped with a temporary filling material (Coltosol F: Coltosol Whaledent, Altstatten, Switzerland), and then incubated at 37 °C. All procedures involving the tooth samples were performed under aseptic conditions in a laminar flow hood (Nuaire, Plymouth, MN, USA).

## Antimicrobial assessment of *E. faecalis* biofilm in infected root samples and teeth slices after intracanal medications

After seven days of medication treatment under the previously described incubation conditions, the temporary filling material was removed. Each root canal and tooth slice were then rinsed with 5 mL of sterile saline (0.09%) to remove any remaining medications [41, 42], followed by an additional wash with 1 mL of 0.5% citric acid solution to neutralize the Ca(OH)_2_ effect. A final irrigation with 3 mL of sterile saline was performed [43]. The remaining bacteria were collected from each root canal sample by gently manually scraping the dentinal walls, 1 mm shorter than the working length (WL), using a K-file #30 (Micro Mega, Besancon, France) for 30 s. This process dislodged the attached biofilms and recovered viable bacteria from the root canal space and dentinal tubules. The intra-canal contents were then collected with paper points, as previously described. The collected paper points and K-file were placed in a vial containing 0.25 mL of sterile saline solution for quantitative evaluation of the bacterial count (S2) using CFU and Q-PCR.

The effect of Ca (OH)_2_ loaded on GA NPs as an intracanal medication on *E. faecalis* biofilm was additionally evaluated through the analysis of its effect on the genes responsible for biofilm formation (*agg, ace, and efaA*). The samples (S_2_) containing isolated RNA from Ca (OH)_2_ loaded on GA NPs treated root canals and untreated samples (control) were analyzed by Q-PCR test to quantify the expression of biofilm genes. Every sample was examined for endogenous (housekeeping Universal16S rRNA) gene: (forward, GACAGGAAAGAAACTAGGAGGAC, reverse, AAACAGACACATCGTGCT) and target biofilm genes (***agg*gene**: forward, TCTTGGACACGACCCATGAT reverse, AGAAAGAACATCACCACGAGC, ***ace*gene**: forward, GAATGACCGAGAACGATGGC reverse, CTTGATGTTGGCCTGCTTCC & ***efaA*** gene: forward, GACAGACCCTCACGAATATG reverse, CCAGTTCATCATGCTGTAGTA) (Invitrogen, Thermo Fisher Scientific, Massachusetts, USA).

The Universal 16 S rRNA gene served as an internal control, and relative amplification efficiency of each gene in each test sample was calculated as follows: (Δ **Ct** control sample = **Ct** target gene – **Ct** endogenous, Δ **Ct** test sample = **Ct** target gene – **Ct** endogenous. The comparative value method was used to calculate the genetic material in each sample (Δ Δ **Ct test** sample = Δ **Ct test** sample – Δ **Ct** control). Then by using the 2- Δ Δ **Ct*(livak)*** method [44], the differential gene expression’s fold change (FC) in comparison to the control was computed. The Fold change indicates whether a gene is up-regulated or down-regulated.

### Scanning Electron Microscope (SEM)

For observational comparison between the study groups, SEM was used to qualitatively evaluate the remaining bacterial biofilm on the teeth slices after treatment with different intracanal medications, as previously described. All the observational analyses for the SEM images were conducted blindly by three different examiners (2nd, 3rd, and 4th authors), each at different times.

## Statistical analysis of the data

Data from Q-PCR and CFU tests were first assessed for normality using the Kolmogorov-Smirnov and Shapiro-Wilk tests. The data exhibited a parametric (normal) distribution, and were thus presented as mean and standard deviation (SD). One-way ANOVA followed by **Tukey’s HSD** test was used to compare more than two groups in unrelated samples. Also, an independent t-test was employed for comparisons between two groups (conventional-sized and nanosized GA and Ca (OH)_2_). Additionally, Two-way ANOVA was used to test to analyze the interaction between variables. The significance level was set at *P* ≤ 0.05, and all statistical analyses were performed using IBM SPSS Statistics Version 20 for Windows.

## Results

### Characterization of intracanal medications

#### Transmission Electron Microscope (TEM) analysis

**Fig. 2:**
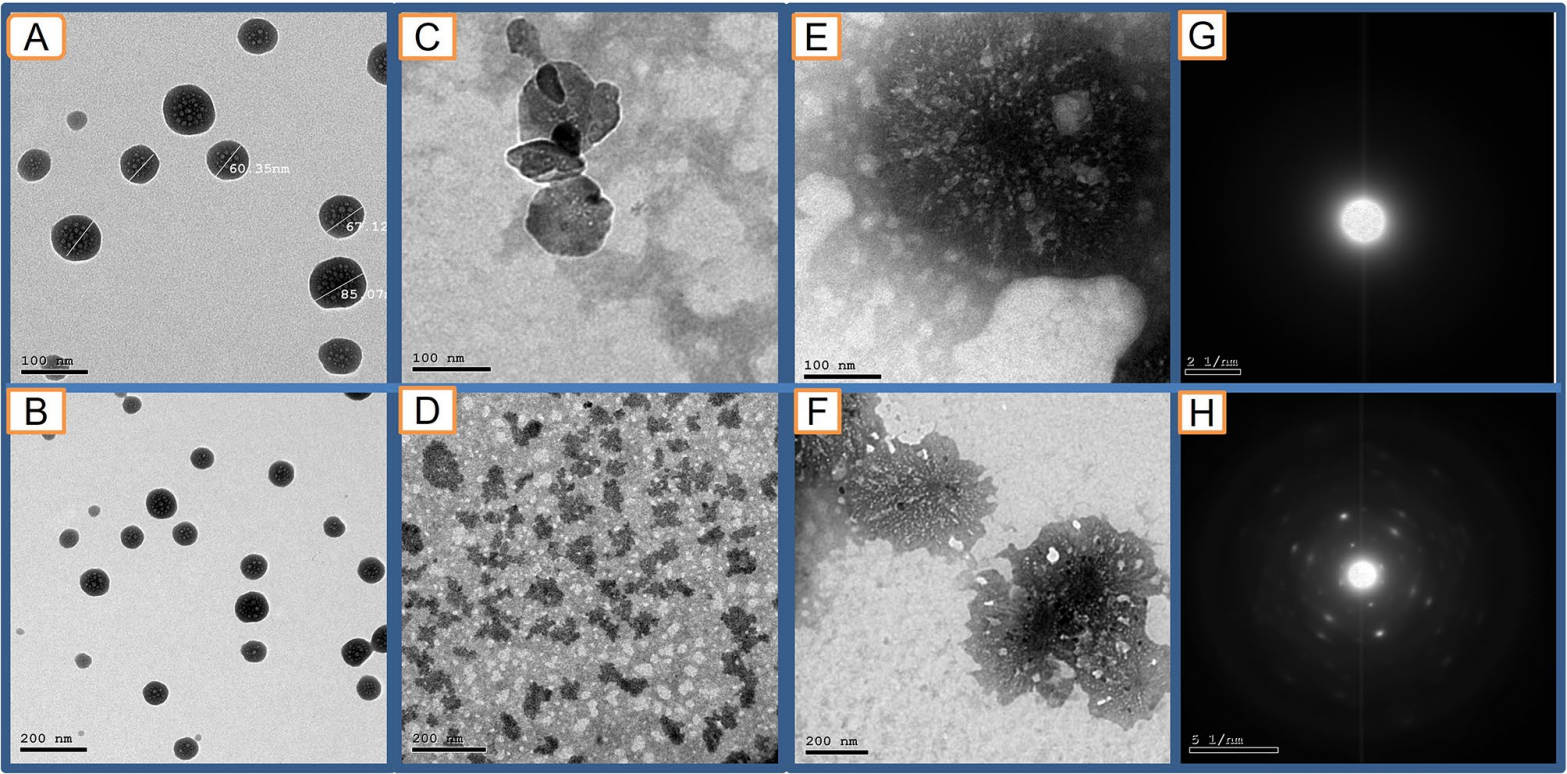
Representative TEM images of the prepared intra-canal medication nanoparticles (NPs) (**A&B**: Ca(OH)_2_ NPs, **C&D**: GA NPs, **E&F**: Ca(OH)_2_ loaded on GA NPs at 100 and 200 nm Magnification, respectively) and SAED images for Ca(OH)_2_ NPs (**G**) and Ca(OH)_2_ loaded GA NPs (**H**)

As described in Fig. 2, TEM images of nano Ca (OH)_2_ showed spherical-shaped pours particles of average size (70.84 nm). Selected Area Electron Diffraction (SAED) showed a uniform nano Ca (OH)_2_ particle distribution. TEM images of Gum-Arabic nanoparticles were mostly spherical, and some oval particles were seen; the average size of the particles was (60.47 nm). The particles showed irregular distribution. Gum-Arabic Nanoparticles did not appear in SEAD as GA is an amorphous polymer. TEM images of Ca(OH)_2_ loaded on GA NPs showed the same amorphous profile oval-shaped particles of GA NPs in addition to the regular concentric distribution of Ca (OH)_2_ particles on the outer surface of GA -NPs of (60.47 nm) size as seen in SEAD.

#### Fourier Transform Infrared Spectroscopy (FTIR) analysis

**Fig. 3:**
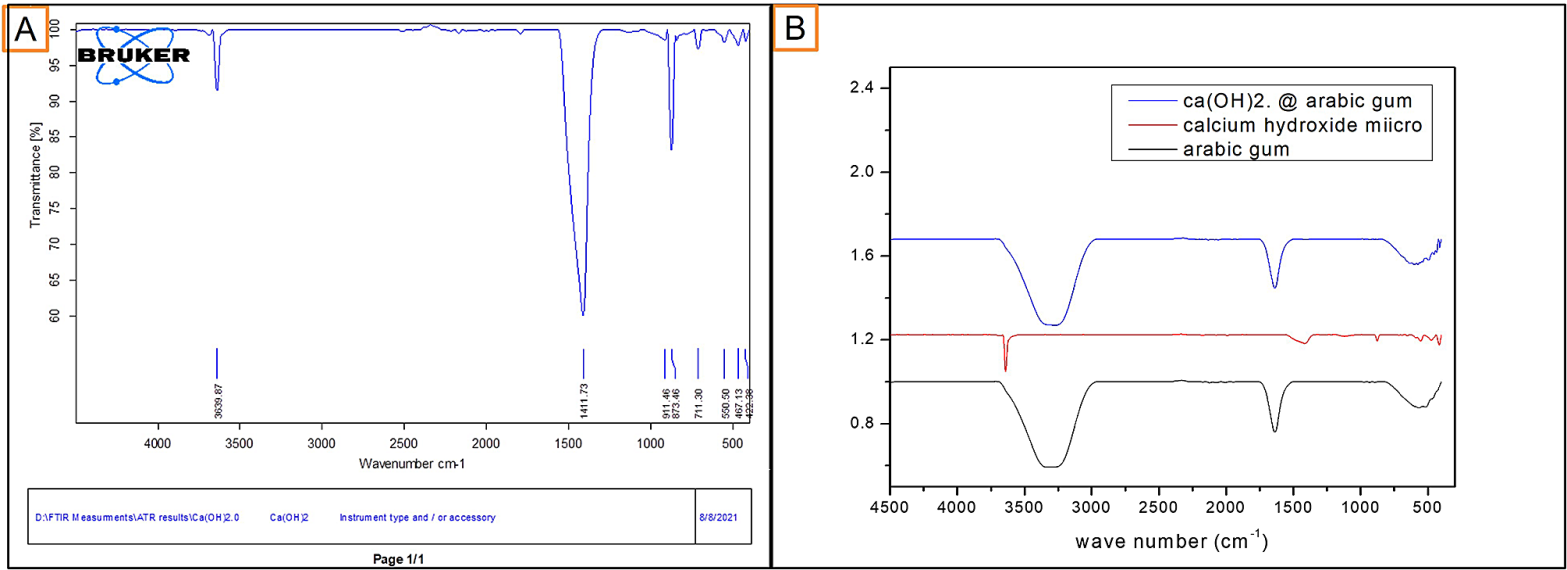
Representative images for FTIR chart of (**A**) calcium hydroxide NPs and (**B**) conventional calcium hydroxide, GA NPs, and calcium hydroxide loaded GA NPs

As described in Fig. 3, FTIR of nano Ca (OH)_2_ showed peaks at wavenumbers 1411.73 cm^− 1^ and 3639.87 cm-1, representing stretching, and bending vibration peaks of the O-H group of nano Ca (OH)_2_. Also, another peak at wavenumbers 873.46 cm^− 1^ corresponded to the symmetric deformation of the carbonate group.

FTIR spectrum of Ca(OH)_2_ showed sharp peaks at 3642 and 1407.43 cm^− 1^ wavenumbers corresponding to the stretching vibration of the O-H group. FTIR spectrum of nano GA showed peaks at 1635 cm^− 1^ and 3413.32 cm-1 wavenumbers related to the asymmetrical extensional vibration of a carboxyl group and extension of the O-H group, respectively. FTIR spectrum of Ca(OH)_2_ loaded on GA-NPs showed Peaks at 1635 cm^− 1^ and 3413.32 cm-1 wavenumbers, similar to the GA-NPs spectrum. Furthermore, no obvious sharp peaks associated with the free drug were detected. These results indicated that neither change in the main backbone of GA NPs polymer structure nor the formation of new bonds between Ca(OH)_2_ and GA-NPs had occurred, referencing a full encapsulation of the Ca(OH)_2_ in the GA-NPs [17, 20].

## Biofilm formation capacity of *E. faecalis*

**Fig. 4:**
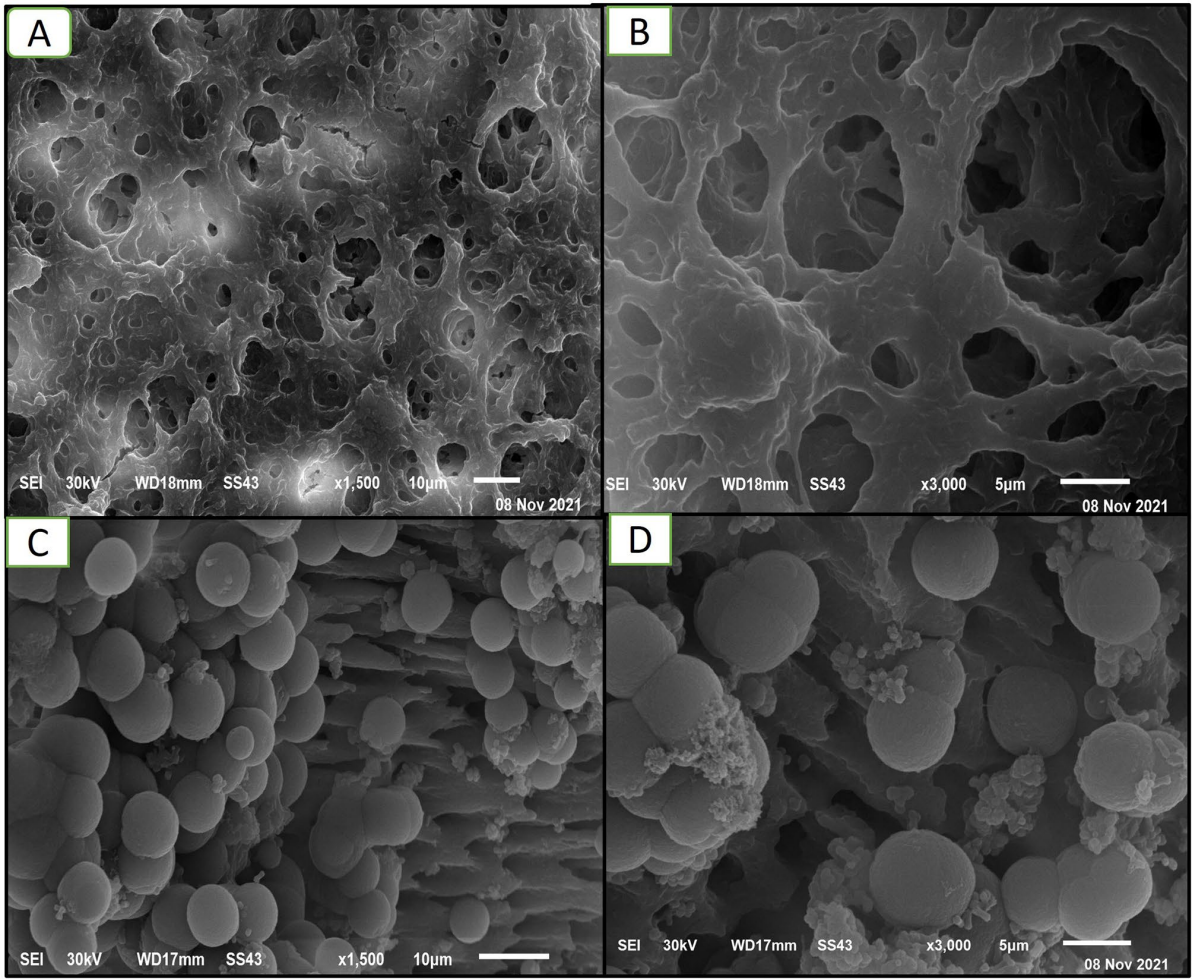
Representative SEM images for the study samples teeth slices; A&B: after samples sterilization (*n* = 1 in each group) and before the *E. faecalis* inoculation, C&D: after *E. faecalis* inoculation and biofilm formation (*n* = 1 in each group) at different magnifications (1.500X &3000X)

Biofilm growth of all *E. faecalis* on the Congo red plate caused blacking of the medium due to the production of exopolysaccharides (EPS), the major constituent of biofilm confirmed biofilm formation, and the plate free from *E. faecalis* remained pink, indicating no biofilm formation (Figure S2). Also, the Scanning Electron Microscopic (SEM) evaluation (Fig. 4) showed the sterilization of root samples before bacterial inoculation, and the formation of *E. faecalis* biofilm on the surface of root canals after *E. faecalis* inoculation was validated in the random samples.

## The effect of tested intracanal medications on *E. faecalis* bacteria

All preoperative samples (S_1_) showed almost the same count, and no sample was excluded from the study.

### Colony Forming Unit (CFU) assessment

**Fig. 5:**
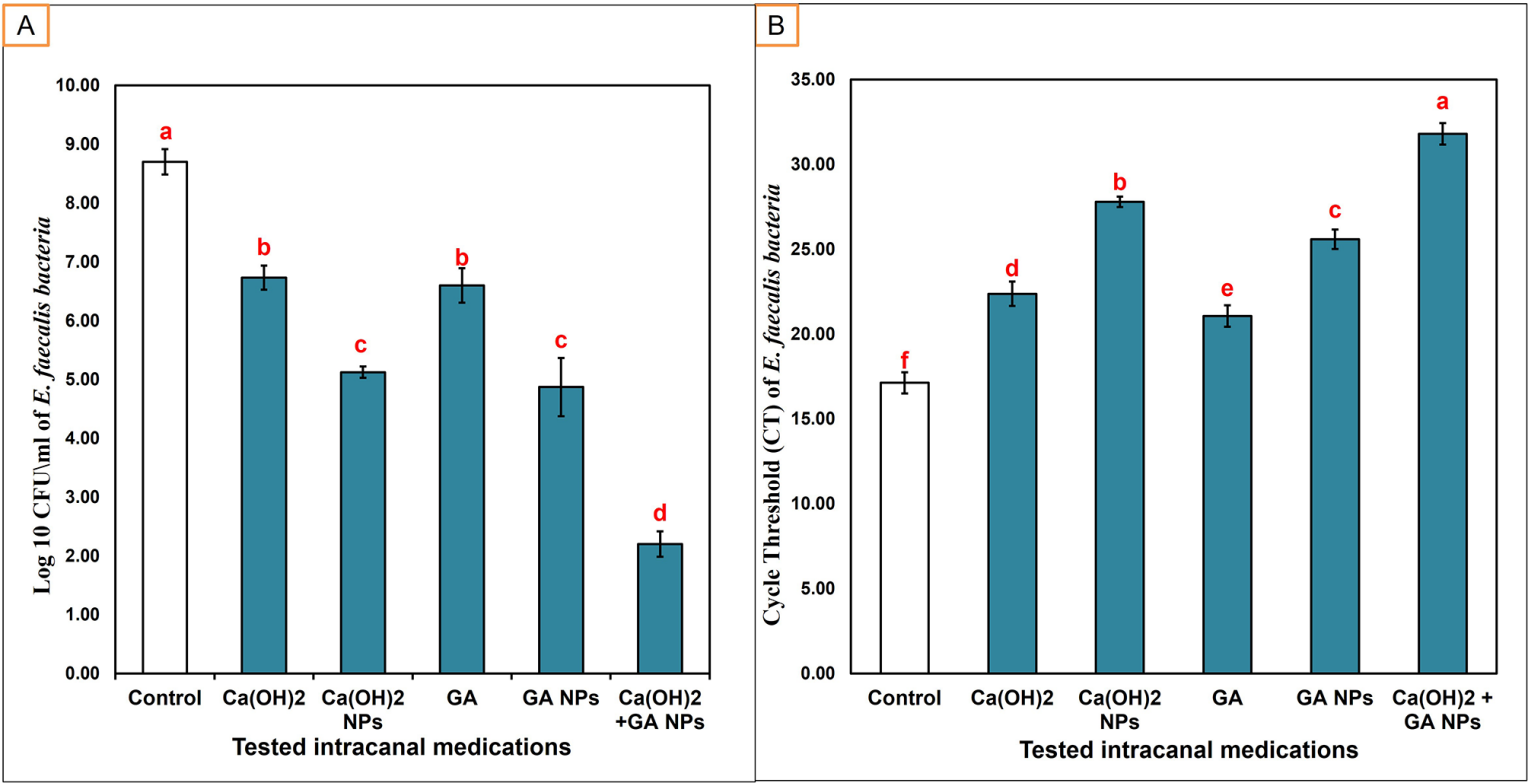
Bar charts representing the assessment of antibiofilm efficacy of the tested intracanal medications, (**A**) bars showing Mean ± SD values for (Log10 CFU/mL) of *E. faecalis* bacteria recovered after tested intracanal medication treatments (*n* = 10 in each group), (**B**) bars showing Mean ± SD values for CT of *E. faecalis* bacteria recovered after tested intracanal medication treatments (*n* = 10 in each group). Means followed by different letters are significantly different according to Tukey’s HSD

Total bacterial counts (Log_10_ CFU/mL) presented as mean and standard deviation were listed in (Supplementary Tables 1,2) and (Fig. 5). The six studied groups showed high statistically significant differences in CFU mean count (*p < 0.001*) except (Ca(OH)_2_, GA groups) and (nano Ca(OH)_2_ and nano GA groups) showed no significant difference with each other. The lowest significant mean total bacterial count was recorded in Ca (OH)_2_ loaded on the GA NPs group (2.20 ± 0.22 CFU/mL). In contrast, the highest significant total bacterial count was recorded in the control group (8.70 ± 0.22 CFU/mL). The Two-way analysis of variance showed an overall highly significant effect (*p < 0.001*). Moreover, there was a highly significant effect induced by Ca (OH)_2_, GA (*p* < 0.001) in NPs formulations when compared to their conventional form (*p < 0.001*).

### Quantitative Polymerase Chain Reaction (Q-PCR) assessment

According to the results of the Q-PCR melting curve (Figure S3), the six studied groups showed high statistically significant differences in the resulting Cycle Threshold (CT) values (p < 0.001***) (Supplementary Tables 3,4) (Fig. 5). The lowest significant mean CT value was recorded in the control group (17.12 ± 0.61) (denoting the highest bacterial count). While the highest significant mean CT value was recorded in Ca (OH)_2_ loaded on the GA NPs group (31.80 ± 0.64) (denoting the lowest bacterial count). The Ca (OH)_2_ and Ca(OH)_2_ NPs revealed more significant CT values than GA and GA NPs, respectively. Two-way analysis of variance showed an overall highly significant effect (*p* < 0.001). Moreover, a highly significant effect was induced by Ca(OH)_2_ and GA in NPs formulations (*p < 0.001*) when compared to their conventional forms.

### Evaluation of the effect of Ca(OH)_2_ loaded on GA NPs on E. faecalis biofilm genes expression

The relative Q-PCR results showed that the mean threshold cycle (CT) values of two examined gene products *(ace and efaA*) were significantly higher in the Ca(OH)_2_ loaded on GA NPs treated samples when compared to the untreated control samples. The resultant mean fold change (FC) for the three tested genes was upregulated (1 ± 0) in the untreated samples, meaning these genes were more expressed. While the mean fold change for *Agg* (0 ± 10), *ace* (0 ± 0), and *efa A* (0 ± 0) genes in the treated samples were downregulated, that means that these genes were less expressed. Statistical data also showed that the fold changes (FC) in the three tested genes’ expression were highly significant (*p* < 0.001) in the treated samples when compared to the control sample (Table 1).

**Table 1:** Showing Mean ± SD values fold changes in expression of biofilm genes (*ace, agg, and efaA*) of *E. faecalis* in the control and Ca(OH)_2_ loaded on GA NPs treated samples

Gene expression		Group				Independent t-test *P* value
		Control (*n* = 10)		Treated (*n* = 10)		
		Mean	±SD	Mean	±SD	
16s rRNA	CT	**26.97**	0.96	24.68	0.71	0.029*
*ace gene*	CT	**24.68**	0.68	30.95	0.90	< 0.001***
	FC	**1.00**	0.00	0.00	0.00	< 0.001***
*Agg* gene	CT	**26.92**	0.85	27.92	0.55	0.162 Ns
	FC	**1.00**	0.00	0.10	0.00	< 0.001***
*efaA* gene	CT	**17.67**	0.61	24.74	0.72	< 0.001***
	FC	**1.00**	0.00	0.00	0.00	< 0.001***

Ns: nonsignificant, *: Significant at *p* < 0.05, ***: significant at *p* < 0.001

### Scanning Electron Microscope (SEM)

**Fig. 6:**
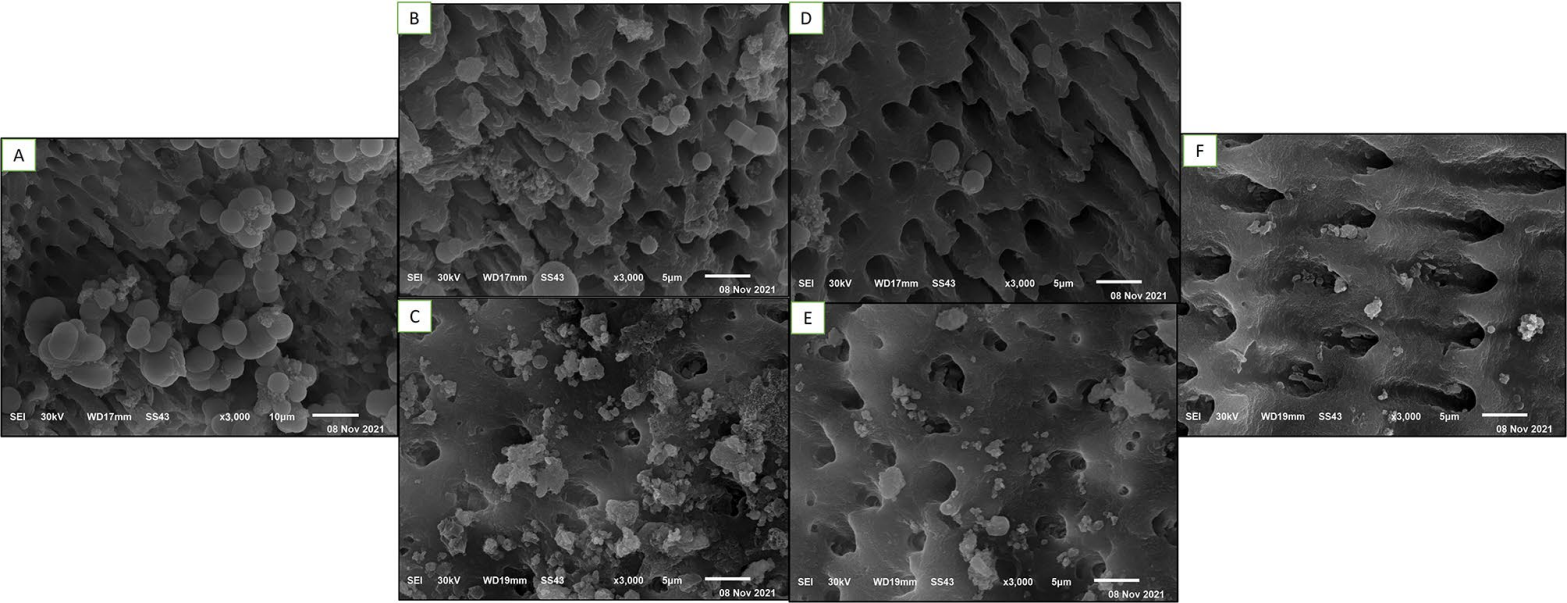
Representative SEM images for *E. faecalis* bacteria response to different intracanal medicament tested (*n* = 5 in each group) **A**: Control (Hydroxypropyl Methyl Cellulose). **B**: Ca (OH)_2_ medication effect, **C**: GA medication effect, **D**: nano Ca (OH)_2_ medication effect, **E**: nano GA medication effect, and **F**: Ca (OH)_2_ loaded on GA NPs medication effect under (x 3000) magnification of SEM. Note that all treatments showed a reduction of bacteria compared to the control one

Regarding the observation analysis for the effect of different intracanal medications on *E. faecalis* biofilm, the biofilm of *E. faecalis* was diminished in all treatment groups except for the control. Moreover, the Ca (OH)_2_ loaded on GA showed greater bacterial reduction than other groups (Fig. 6).

## Discussion

Resistant and persistent bacteria after chemo-mechanical preparation inside the root canal system is considered an endodontic nightmare. The presence of complex root canal anatomy acts as a favorable shelter for the growth of *E. faecalis* bacteria [2–4]. The small size of *E. faecalis* enables it to rapidly invade and colonize inside dentinal tubules, isthmi, ramifications, or recesses and hide from conventional medications [45]. Despite the excellent properties of Ca(OH)_2_ as an intracanal medication, it was reported to have certain weaknesses against *E. faecalis* [46–48]. To solve the challenges stated above, various disinfection protocols have been proposed to increase the success rate of root canal treatment.

Because of the improved antimicrobial effect of medicaments in nanoparticles [14–16], studies have suggested either their combination with conventional medicaments or using nanoparticles as a carrier for intracanal medicaments delivery [49]. The recent world trend is directed toward medication encapsulated into natural polymeric nanocarriers because of their intrinsic biocompatibility, biodegradability, and higher antibacterial activity [22, 50–54]. Accordingly, the current in-vitro study used the developments in nanotechnology and natural polymer synthesis to introduce Ca(OH)_2_ loaded on GA nanocarrier as a new intracanal medication formulation.

The new nanoparticle formulation was characterized using TEM to evaluate the size, shape, and distribution of prepared NPs. Fourier-transform infrared Spectroscopy (FTIR) was done to evaluate the compatibility of prepared medications by observing functional groups involved in stabilizing synthesized nanoparticles [20, 55]. The absence of peaks indicative of free Ca(OH)_2_ in the Ca(OH)_2_ loaded GA nanocarrier indicated that it was not a mixture of both Ca(OH)_2_ and GA in the same sample [17]. Instead, Ca(OH)_2_ was successfully encapsulated inside the GA nanocarrier particles. This encapsulation was important to ensure that the pharmacokinetics of loaded Ca(OH)_2_ were not affected and that no formation of new compounds with unknown biological behavior had occurred [17, 20].

It was important in the current study to evaluate whether the newly introduced intracanal medication formulation could give more anti-*E. faecalis* biofilm efficacy compared to the other tested medications, including the conventionally used calcium hydroxide or not. No previous study was found to assess the antibacterial efficacy of Ca(OH)_2_ and GA comparatively, either in their conventional or nanoparticle size. Therefore, the Ca(OH)_2_ loaded on GA nanocarrier, GA, nano GA, Ca(OH)_2_, and nano Ca(OH)_2_ antimicrobial efficacy against mature *E. faecalis* biofilm inside infected root canals was also compared.

An in vitro endodontic infection model using *E. faecalis* standard strain collection (ATCC #29,212) was used in the current study, as this bacterium was found to be most commonly associated with failed root canal treatment [56]. Also, most disinfection studies used *E. faecalis* in evaluating the antibacterial efficiency of different intracanal medications. So, as the current study used new intracanal medication formulations, we could compare the results with the previous studies [36, 57].

To get predictable outcomes in this study, different laboratory models of dentinal tubule infection and evaluation tools for the antibacterial effect of the tested intracanal medications on *E. faecalis* biofilm were used [57]. According to previous endodontic disinfection studies, the root canal model was used to closely mimic the clinical conditions [36, 57]. SEM evaluation was also used in different studies to monitor *E. faecalis* properties, including its aggregation, invasion into dentinal tubules, and biofilm morphological and structural characterization [2, 36, 57]. Since the SEM visualization of the formed biofilm could not be done for the root canal model without root sectioning, that could detach and affect the formed biofilm structure. So, in the current study, additional separate tooth slice models were prepared for observational analysis of bacterial reduction in response to different tested medications using high-resolution SEM images [57].

The coronal part of root dentine was used to prepare standardized tooth slices because it has a more uniform and greater diameter of dentinal tubules (4.32 μm) with less inter-tubular dentine than in the apical zone (1.73 μm) [55, 58]. This allows more area for bacterial invasion during inoculation and better inspection using SEM analysis. In contrast, the apical dentine has marked structural variations, including irregular dentinal tubules in both direction and density, varied amounts of irregular secondary dentine (areas might be devoid of tubules), accessory root canals, areas of resorption, pulp stones, and even cementum-like tissue lining the apical root canal wall [59, 60].

Previously, it was shown that, the biofilm of refractory endodontic infections, including *E.faecalis* biofilm, survived under nutritionally stressful environmental conditions inside the root canals. This possibly renders them resistant to the chemo-mechanical disinfecting procedures [61, 62]. Thus, in the current study, to mimic this clinical condition, the frequency of refreshing the culture medium was limited to three-day intervals instead of daily medium change as a way to increase the bacterial biofilm resistance to antimicrobial treatments [39, 57, 62].

For quantitative evaluation, CFU was used as a simple method for counting bacteria, but it could only discover bacteria that could start cell division at a rate fast enough to establish colonies. To overcome these drawbacks, Q-PCR was also used to compare the antibiofilm efficiency of different medications due to its ability to identify and quantify one or more particular sequences in a gene sample [63]. Q- PCR relies on DNA’s enzymatic replication and thermal cycling. Consequently, estimating the amount of target gene copies in a sample has been utilized to estimate and compute bacterial aggregations in biofilms. Compared to traditional PCR, Q-PCR was reported to be more accurate and sensitive [57, 63]. Besides, to focus on the antibiofilm efficacy of the newly formulated Ca(OH)_2_ loaded on GA NPs medication, its effect on the expression of biofilm-forming genes (*ace, agg, and efaA*) using relative Q-PCR was also analyzed.

The colonization of dental root surfaces by *E. faecalis* is enhanced by the expression of adhesion factors (like *ace* and *agg* genes) through increasing their adhesion to dentinal collagen [64]. The *ace* gene was shown to enhance the adherence of *E. faecalis* to human collagen type IV and human dentine [65]. Besides, the aggregation genes have been shown to promote *E.faecalis* adhesion to the host cell through extracellular matrix protein adherence and elevated cell surface hydrophobicity. It was found that the *E.faecalis* that encoded the *agg* gene resisted phagocytosis by inhibiting the respiratory burst in macrophages, which results in the generation of reactive oxygen species (ROS) [66]. Also, the growth of *E. faecalis* in serum-containing media has been shown to promote the expression of endocarditis virulence factor (*efaA*). Since cardiac and dentinal tissues both contain the same proteins, *efaA* has been shown to improve the attachment of *E. faecalis* to collagen and extracellular matrix in root canal infections [67–69].

In the current study, the null hypothesis was partially rejected. All the tested medications demonstrated a significant antimicrobial effect against *E. faecalis* compared to the control (untreated samples). Ca(OH)_2_ and Ca(OH)_2_ NPs showed comparable antibacterial effects to GA and GA NPs, respectively, when the CFU test was used. On the other hand, when using the Q-PCR test, there were significant differences between all the tested intracanal medications regarding their antibacterial efficacy. The difference in the Q-PCR and CFU test results emphasizes the importance and sensitivity of the molecular methods in determining the bacterial count [57].

Ca(OH)_2_ and its NPs showed a greater anti-*E. faecalis* effect than GA and its NPs. In the literature, there were contradictory results about the sensitivity of *E. faecalis* to Ca(OH)_2_ [10–12, 70–75]. Some studies [9, 71–73] agreed with the current results and supported the presence of Ca(OH)_2_ antibacterial effect against *E. faecalis.* It was demonstrated that most microorganisms could not survive in excessively alkaline environments like that of Ca(OH)_2_ (12.5–12.8) [76]. Ca (OH)_2,_ when contacted with an aqueous solution, breaks down into calcium and hydroxyl ions. These ions cause the major antibacterial actions of Ca(OH)_2_ on vital tissues [9].

On the other hand, other studies showed a reduced Ca(OH)_2_ antibacterial effect against *E. faecalis* when compared to other antibacterial agents (like Liquorice extract, Propolis, and triple antibiotic mixture) [10, 12, 74]. Also, a prominent resistance of *E. faecalis* against Ca(OH)_2_ was reported to be due to their proton pump activity [70, 75], which causes the environment to become acidic, and its biofilm-forming ability. Recently, it was also shown that Ca(OH)_2_ could help in *E. faecalis*’s biofilm calcification [48]. These factors enable *E. faecalis* to overcome the high pH levels of Ca(OH)_2_, augmented by the dentine buffering action [46, 47]. The contradiction in the results between studies might also be due to the variation in bacterial organization within the biofilm’s extracellular polymeric substance (EPS) and different biofilm substrates used that significantly impacted the Ca(OH)_2_ antibacterial efficiency.

The resulting GA antibacterial effect in the current study was in accordance with previous studies [23, 26, 77–81] and was attributed to its great salt composition of Ca^+ 2^, Mg^+ 2,^ and K^+ 2^ of polysaccharides and its efficacy in the metabolism of Ca and possibly phosphate [77, 78]. Also, numerous types of enzymes like peroxidases, oxidases, and pectinases were found in the GA composition, some of which have antibacterial effects [79]. The antibacterial efficacy of GA has been additionally linked to their secondary metabolites or the presence of flavonoids, alkaloids, phenol, volatile oil, saponin, saponin glycosides, triterpenoid, and hydrolyzable tannin [80, 81].

Both tested materials (Ca (OH)_2_ and GA) nanoparticles in the current study had better antibacterial and antibiofilm efficiency than their conventional form. Similarly, other studies have shown that using Ca(OH)_2_-NPs could effectively penetrate the dentinal tubules deeper than conventional Ca (OH)_2_, causing greater efficacy in the *E. faecalis* biofilm eradication from dentinal tubules [14–16]. The higher antibacterial efficiency might be attributed to the change in the physiochemical properties of the materials when they are prepared in nanosized [82]. Since the nanoparticles have more chemical and biological reactivity than their conventional counterparts [49], they could interact with negatively charged bacterial cells more due to their larger surface area and higher charge density [50].

In the present study, Ca(OH)_2_ was loaded on GA NPs as a nanocarrier, aiming to increase the antibacterial efficiency of Ca(OH)_2_. Fortunately, this new formulation showed the greatest antibiofilm efficiency in comparison to all other tested medications in all the tests used in the current study (CFU, Q-PCR, SEM). This was in agreement with previous studies that revealed that combining calcium hydroxide with silver nanoparticles or zinc oxide nanoparticles was more effective in eliminating *E. faecalis* from infected dentine than conventional Ca(OH)_2_ alone [50, 83, 84]. Nagarathinama et al. [55], in their study loaded the triple antibiotic past on apatitic nanocarriers (TAAN) and showed significantly higher antibacterial biofilm against *E. faecalis* than conventional TAP. In another study by Arafa et al. [85], ciprofloxacin (CIP) was loaded on PLGA nanoparticles and showed significantly higher antibacterial biofilm against *E. faecalis* than conventional ciprofloxacin. On the contrary, Farhadian et al. [17] found that the *E. faecalis* inhibition zones around Gum Tragacanth polymer, Ca(OH)_2_ and Ca(OH)_2_ encapsulated with Gum Tragacanth disks were similar when agar disc diffusion method was used.

The significant decrease in the biofilm-forming genes (*ace, agg, and efaA*) detected by relative Q-PCR when *E. faecalis* was treated with Ca (OH)_2_ loaded on GA NPs compared with the untreated sample (control) was in agreement with a previous study [86]. This study showed that using chitosan-propolis nanoparticles in biofilm eradication increases the antibacterial efficiency by altering the expression of *E*. *faecalis* biofilm-associated genes [86].

The increased antibacterial effect of Ca (OH)_2_ loaded on GA NPs in the current study was probably due to favorable hygroscopic compatibility, attracting electrostatic force and synergism between GA NPs polymeric matrix and Ca (OH)_2_. Additionally, it could be explained by the NPs’ lesser size (below 200 nm) than the dentinal tubules’ diameter, which ranges from 2400 to 4280 nm [87]. So, it could be predicted that Ca(OH)_2_ and GA medicines would be delivered deeper into the dentinal tubules and biofilm. Thus avoiding the dentinal tubules’ buffering action and increasing their antibacterial effect [20, 51]. Similarly, a previous study [20] showed a marked greater Ca(OH)_2_ depth and area of penetration into dentinal specimens when loaded into poly lactic-co-glycolic acid (PLGA) nanocarrier, in comparison to conventional Ca (OH)_2_. Furthermore, it was shown previously that nanocarriers could deliver high drug concentrations for a long time [52]. Thus, the sustained Ca(OH)_2_ release by GA nanocarrier could be another reason for increasing its efficiency in invading biofilm [17, 19, 51].

Despite the promising results from CFU, Q-PCR, and SEM tests regarding the new intracanal medicament formulation in the current study, testing *E*. *faecalis* virulence genes in response to the other tested medications is suggested in further studies. Additional cytotoxicity evaluation for the tested medications in the current study is highly recommended to achieve a more decisive conclusion regarding this new formulation.

Another limitation is that this study was done in vitro, using only one type of bacteria in the endodontic infection model. This did not reflect the in-vivo conditions, including the synergism between different bacterial types, buffering oral environment, and the bacterial phenotype diversity in the endodontic infection. Testing the antibacterial effect of these medications, including a wider range of intra-canal microorganisms, using more advanced molecular identification methods like next-generation sequencing is recommended. Thus, the current study could reveal promising results for Ca (OH)_2_ loaded on GA NPs formulation, suggesting new root canal disinfection protocols, especially in regenerative endodontics. However, this formulation requires more research representing the clinical state in further in-vivo studies.

## Conclusions

Within the limitations of the present study, it is concluded that the Ca(OH)_2_ NPs and GA NPs have superior antimicrobial and antibiofilm activity against *E. faecalis* compared to conventional Ca (OH)_2_ and GA. Furthermore, Ca(OH)_2_ loaded on GA-NPs showed the best antimicrobial and antibiofilm efficacy in treating *E. faecalis* biofilm-infected root canals and it could be considered a promising intracanal medication instead of conventional Ca(OH)_2_.

### Electronic supplementary material

Below is the link to the electronic supplementary material.

Supplementary Material 1: Figure S1. The PRILE flowchart [28] for the current study

Supplementary Material 2: Figure S2. Photomicrograph showing A: growth of *E. faecalis* on CRA caused blacking of the medium due to EPS production referring to biofilm formation and (B) no change in color in the control sample

Supplementary Material 3: Figure S3. Representative image for Q-PCR melting curve showing CT of *E. faecalis* bacteria in response to different tested intracanal medications; (c) representing control group, (1) representing Ca (OH)_2_, (2) representing nano Ca (OH)_2_, (3) representing GA, (4) representing nano GA, (5) representing Ca (OH)_2_ loaded on nano GA

Supplementary Material 4

## Data Availability

The datasets generated and/or analyzed during the current study are not publicly available due to privacy but are available from the corresponding author upon reasonable request.

## Abbreviations

Ca(OH)_2_: Calcium hydroxide
GA: Gum Arabic
NPs: Nano-particles
REC: Research Ethics Committee
TEM: Transmission Electron Microscope
FTIR: Fourier-Transform Infrared Spectroscopy
CFU: Colony Forming Unit
Q-PCR: Quantitative Polymerase Chain Reaction
SEM: Scanning Electron Microscope
CT: Cycle Threshold
EDTA: Ethylenediaminetetraacetic acid
NaOCl: Sodium hypochlorite
