# Supplementary figures and images for: Antibacterial biofilm efficacy of calcium hydroxide loaded on Gum Arabic nanocarrier: an in-vitro study
Source: BMC Oral Health. 2024 Feb 10;24:215. doi: 10.1186/s12903-024-03941-3 (PMC10859034; doi:10.1186/s12903-024-03941-3)

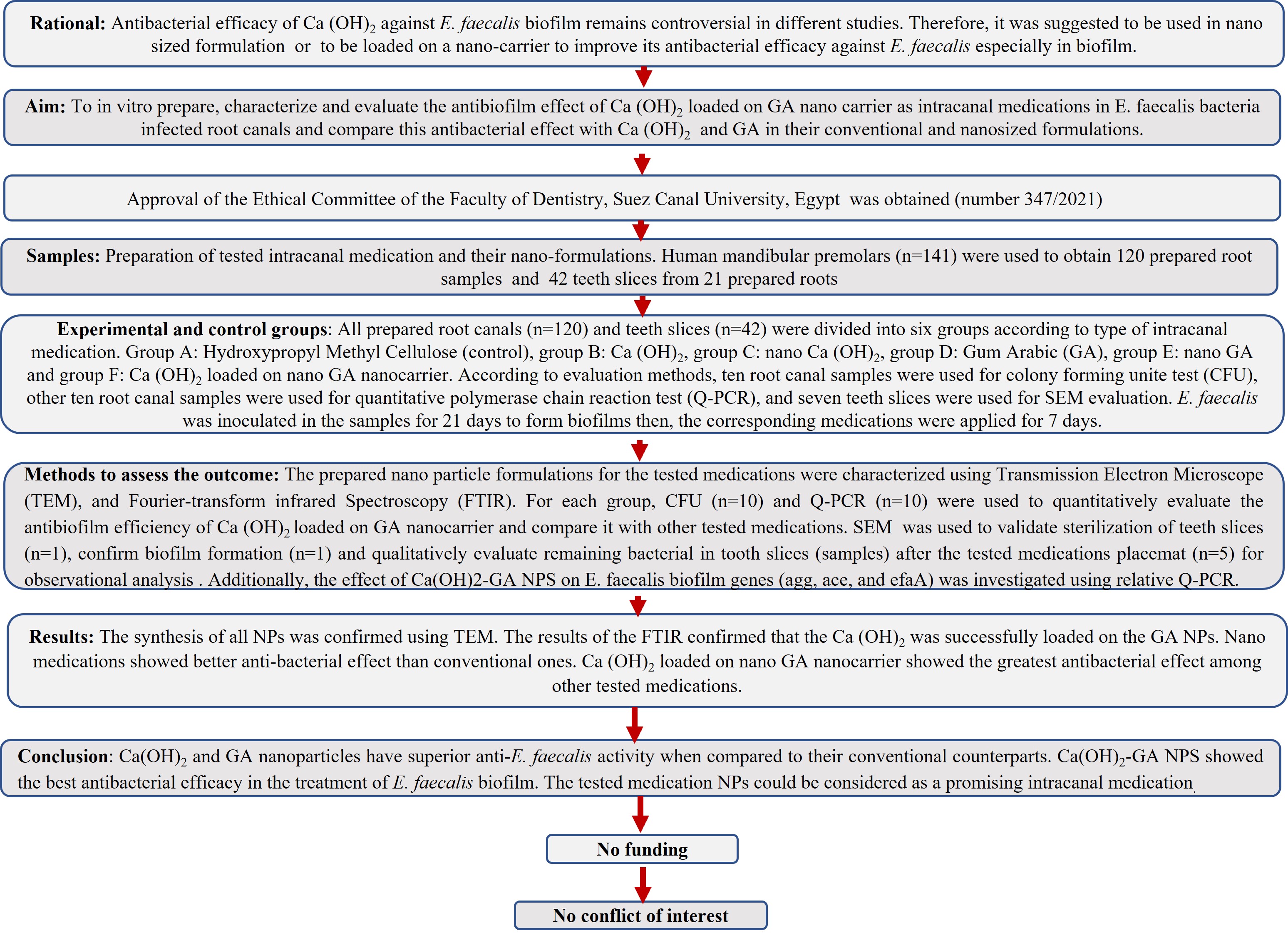

Supplement: Supplementary file 1 — Supplementary Material 1: Figure S1. The PRILE flowchart [28] for the current study [file 12903_2024_3941_MOESM1_ESM.jpg]

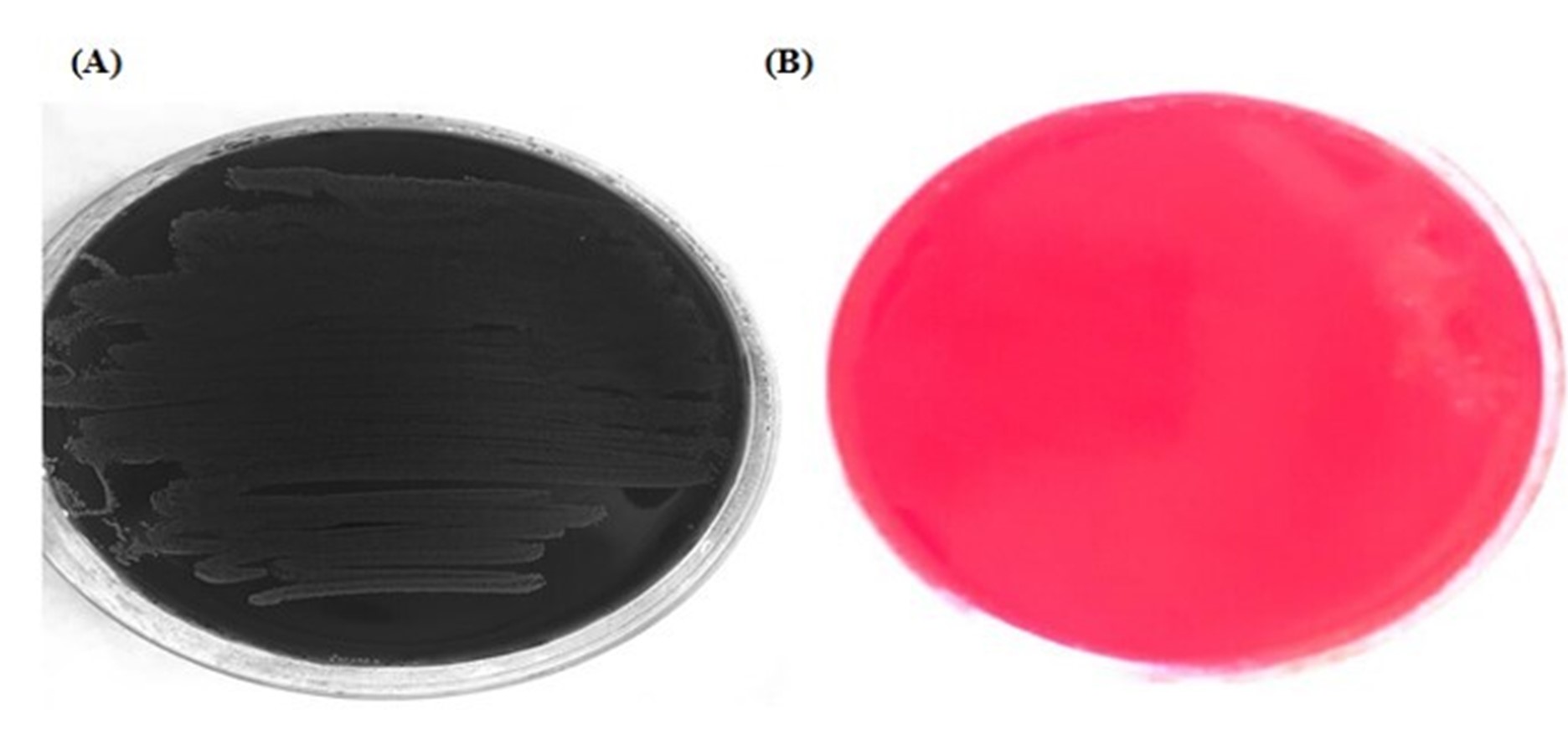

Supplement: Supplementary file 2 — Supplementary Material 2: Figure S2. Photomicrograph showing A: growth of E. faecalis on CRA caused blacking of the medium due to EPS production referring to biofilm formation and (B) no change in color in the control sample [file 12903_2024_3941_MOESM2_ESM.jpg]

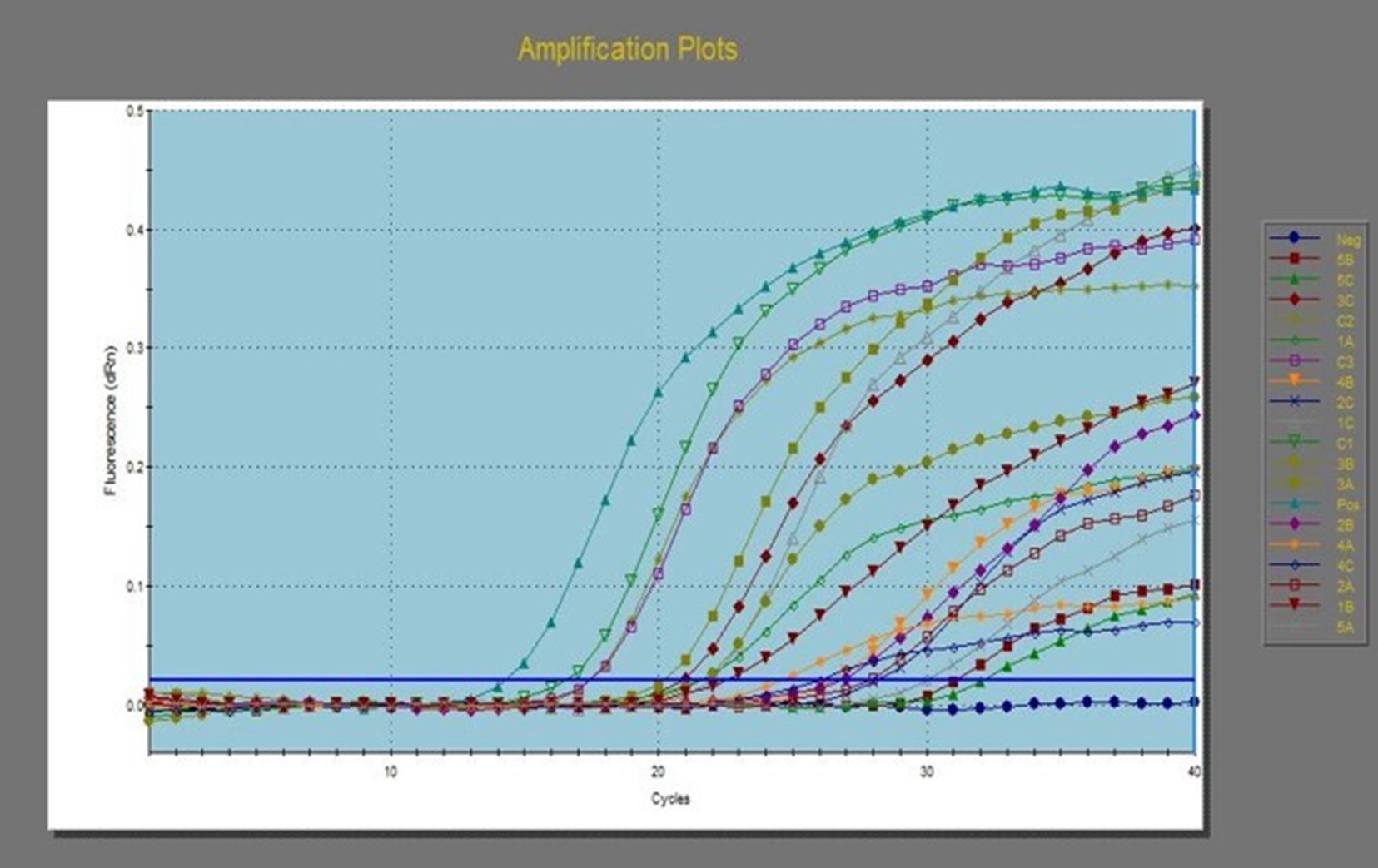

Supplement: Supplementary file 3 — Supplementary Material 3: Figure S3. Representative image for Q-PCR melting curve showing CT of E. faecalis bacteria in response to different tested intracanal medications; (c) representing control group, (1) representing Ca (OH)2, (2) representing nano Ca (OH)2, (3) representing GA, (4) representing nano GA, (5) representing Ca (OH)2 loaded on nano GA [file 12903_2024_3941_MOESM3_ESM.jpg]
