# Supplementary material for: Antibacterial biofilm efficacy of calcium hydroxide loaded on Gum Arabic nanocarrier: an in-vitro study
Source: BMC Oral Health. 2024 Feb 10;24:215. doi: 10.1186/s12903-024-03941-3 (PMC10859034; doi:10.1186/s12903-024-03941-3)
Supplement: Supplementary file 4 — Supplementary Material 4 [file 12903_2024_3941_MOESM4_ESM.docx]

**Supplementary table 1.** Showing Mean ±SD values of the effect of different tested medications Ca (OH)_2_, Ca (OH)_2_ NPs, GA, GA NPs, Ca (OH)_2_ loaded on GA NPs on E. faecalis bacteria Log_10_ CFU/ml.

| **Group** | **Log_10_ CFU/ml** | | |
| --- | --- | --- | --- |
|  | **Mean** | ±**SD** | **Tukey’s HSD** |
| **Control** | 8.70 | 0.22 | A |
| **Ca (OH)_2_** | 6.73 | 0.21 | B |
| **Ca (OH)_2_ NPs** | 5.27 | 0.25 | C |
| **GA** | 6.60 | 0.29 | B |
| **GA NPs** | 4.40 | 0.29 | C |
| **Ca (OH)_2_ loaded GA NPs** | 2.20 | 0.22 | D |
| **ANOVA** | | | |
| **Source** | **F** | | **Sig.** |
| **Corrected Model** | 808.0 | | <.001*** |
| **Intercept** | 30966.6 | | <.001*** |
| **Ca (OH)_2_** | 701.8 | | <.001*** |
| **GA** | 1079.1 | | <.001*** |
| **NPs** | 543.2 | | <.001*** |

Means followed by different letters within the same column are statistically significant at p<0.001, according to **Tukey’s HSD.** *** denotes high significant effect at p<0.001

**Supplementary table 2.** Showing Mean ±SD values of the effect of conventional and nano medications on E. faecalis bacteria Log_10_ CFU/ml.

| **Group** | **Log10 CFU/ml** | | **Independent t-test** | |
| --- | --- | --- | --- | --- |
|  | **Without NPs** | **with NPs** | **T** | **p-value** |
| **Control** | 8.70 ± 0.22 | --- | --- | --- |
| **Ca (OH)_2_** | 6.73 ± 0.21 | 5.27 ± 0.25 | 14.35 | <0.001*** |
| **GA** | 6.60 ± 0.29 | 4.40 ± 0.29 | 16.71 | <0.001*** |
| **Ca (OH)_2_ loaded on GA NPs** | --- | 2.20 ± 0.22 | --- | --- |
| **ANOVA** | **<0.001***** | **<0.001***** |  |  |
| **Source** | **F** | **Sig.** |  | |
| **Corrected Model** | 808.0 | <.001*** |  |  |
| **Intercept** | 30966.6 | <.001*** |  |  |
| **Ca (OH)_2_** | 701.8 | <.001*** |  |  |
| **GA** | 1079.1 | <.001*** |  |  |
| **NPS** | 543.2 | <.001*** |  |  |

*** means that comparisons within the same row (between both Ca (OH)_2_  &Ca (OH)_2_ NPs in addition to the comparison between both GA & GA NPs) are statistically significant different at p<0.001 according to Independent t-test

**Supplementary table 3**. Showing Mean ±SD values for CT of E. faecalis bacteria in response to different tested intracanal medications.

| **Group** | **CT** | | |
| --- | --- | --- | --- |
|  | **Mean** | **±SD** | **Tukey’s HSD** |
| **Control** | 17.12 | 0.61 | F |
| **Ca (OH)_2_** | 22.37 | 0.72 | D |
| **Ca (OH)_2_ NPs** | 27.79 | 0.31 | B |
| **GA** | 21.07 | 0.63 | E |
| **GA NPs** | 25.59 | 0.57 | C |
| **Ca (OH)_2_ loaded GA NPs** | 31.80 | 0.64 | A |
| **ANOVA** | | | |
| **Source** | **F** | | **Sig.** |
| **Corrected Model** | 768.3 | | <.001*** |
| **Intercept** | 100157.5 | | <.001*** |
| **Ca (OH)_2_** | 927.9 | | <.001*** |
| **GA** | 447.4 | | <.001*** |
| **NPS** | 699.6 | | <.001*** |

Means followed by different letters within the same column are statistically significant at p<0.001, according to **Tukey’s HSD.** *** denotes high significant effect at p<0.001

**Supplementary table** 4. Showing Mean ±SD values for CT of E. faecalis bacteria in response to conventional and nano particles intracanal medications.

| **Group** | **CT** | | **Independent t-test** | |
| --- | --- | --- | --- | --- |
|  | **Without NPs** | **with NPs** | **t** | **p-value** |
| **Control** | 17.12 ± 0.61 | --- | --- | --- |
| **Ca (OH)_2_** | 22.37 ± 0.72 | 27.79 ± 0.31 | -21.98 | <0.001*** |
| **GA** | 21.07 ± 0.63 | 25.59 ± 0.57 | -16.70 | <0.001*** |
| **Ca (OH)_2_ loaded GA NPs** | --- | 31.80 ± 0.64 | --- | --- |
| **ANOVA** | **<0.001***** | **<0.001***** |  |  |
| **Source** | **F** | **Sig.** |  | |
| **Corrected Model** | 768.3 | <.001*** |  |  |
| **Intercept** | 100157.5 | <.001*** |  |  |
| **Ca (OH)_2_** | 927.9 | <.001*** |  |  |
| **GA** | 447.4 | <.001*** |  |  |
| **NPS** | 699.6 | <.001*** |  |  |

*** means that comparisons within the same row (between both Ca (OH)_2_  &Ca (OH)_2_ NPs in addition to the comparison between both GA & GA NPs) are statistically significant different at p<0.001 according to Independent t-test.
